# Investigation of biological activity of soil fungal extracts and LC/MS-QTOF based metabolite profiling

**DOI:** 10.1038/s41598-021-83556-8

**Published:** 2021-02-26

**Authors:** Afrah E. Mohammed, Hana Sonbol, Suaad Saleh Alwakeel, Modhi O. Alotaibi, Sohailah Alotaibi, Nouf Alothman, Rasha Saad Suliman, Hanadi Talal Ahmedah, Rizwan Ali

**Affiliations:** 1 Department of Biology, College of Science, Princess Nourah Bint Abdulrahman University, Riyadh, 84428 Saudi Arabia; 2 Pharmaceutical Sciences Department, College of Pharmacy, King Saud Bin Abdulaziz University for Health Sciences, Riyadh, Saudi Arabia; 3 King Abdullah International Medical Research Center, City, Riyadh, Saudi Arabia; 4 Department of Medical Laboratory Technology, Faculty of Applied Medical Sciences, King Abdulaziz University, Rabegh, Saudi Arabia; 5 Medical Research Core Facility and Platforms, King Abdullah International Medical Research Center, King Saud Bin Abdulaziz University for Health Sciences, Ministry of National Guard Health Research, Riyadh, 11481 Kingdom of Saudi Arabia

**Keywords:** Drug discovery, Antibiotics

## Abstract

Soil is considered an extensively explored ecological niche for microorganisms that produce useful biologically active natural products suitable for pharmaceutical applications. The current study aimed at investigating biological activities and metabolic profiles of three fungal strains identified from different desert sites in Saudi Arabia. Soil fungal isolates were collected from AlQasab, Tabuk, and Almuzahimiyah in Saudi Arabia and identified. Furthermore, their antibacterial activity was investigated against *Staphylococcus aureus, Enterococcus faecalis, Klebsiella pneumonia,* and *Escherichia coli* in blood, nutrient, and Sabouraud dextrose agars. Moreover, fungal extracts were evaluated on cell viability/proliferation against human breast carcinoma and colorectal adenocarcinoma cells. To identify the biomolecules of the fungal extracts, High-performance liquid chromatography HPLC–DAD coupled to analytical LC–QTOF-MS method was employed for fungal ethyl acetate crude extract. Identified fungal isolates, *Chaetomium* sp. *Bipolaris* sp. and *Fusarium venenatum* showed varied inhibitory activity against tested microbes in relation to crude extract, microbial strain tested, and growth media. *F. venenatum* showed higher anticancer activity compared to *Chaetomium* sp*.* and *Bipolaris* sp*.* extracts against four of the tested cancer cell lines. Screening by HPLC and LC/MS-QTOF identified nine compounds from *Chaetomium* sp. and three from *Bipolaris* sp. however, for *F. venenatum* extracts compounds were not fully identified. In light of the present findings, some biological activities of fungal extracts were approved in vitro*, s*uggesting that such extracts could be a useful starting point to find compounds that possess promising agents for medical applications. Further investigations to identify exact biomolecules from *F. venenatum* extracts are needed.

## Introduction

Soil is a rich source of different microorganisms that tolerate the environmental changes via the synthesis of natural products as survival strategies to handle extreme habitat. Microbes from harsh soil like deserts with exceptional environmental conditions could provide unique metabolites that serve as potential pharmaceutical products; however, some are detrimental^1^. Amongst microbes, fungi were the most abundant natural product producers, estimated at 42%^2^. Therefore, fungi could be considered one of the major constituents of microbial production industries since their metabolites could be an alternative for health security. Metabolites are low molecular weight compounds associated with several potentially beneficial biological activities^3,4^. Fungi inhabit harsh habitats and produce unique secondary metabolites, including cryoprotectant compounds such as sugars and polyols to sustain turgor pressure of the membranes. Osmotically active molecules polyols produced by xerotolerant species, and fungal melanins pigment serve as a shield against UV radiation and frizzing^5–7^.

Recently, the increasing rate of bacterial resistance to antibiotics has led to seeking other microbial origins capable of overcoming microbes in the clinical and agriculture field^8^. Antibiotics extracted from microorganisms inhabiting extreme conditions such as drought, strong acidic or alkaline pH, and temperature extremes are likely to have unique features^9^. Furthermore, many clinical secondary metabolites are extracted from fungi, such as the β‐lactam antibiotics, penicillin, cephalosporin, griseofulvin, and the ergot alkaloids^10^. A recent study by Gashgari et al.^11^ showed the antimicrobial ability of fungal extracts isolated from medicinal plants in Saudi Arabia against important human pathogenic bacteria. For example, *Penicillium chrysogenum, Fusarium oxysporum,* and *Fusarium nygamai* showed strong antibacterial activity against *Staphylococcus aureus, Escherichia coli, Pseudomonas aeruginosa*, and *Klebsiella pneumoniae*.

On the other hand, in 2018, the estimated number of new cancer cases worldwide was about 17 million, with 9.6 million cancer-related deaths and new cases areare expected to be 27.5 million by 2040^12^. Lung, colorectal, stomach, liver, and breast cancers are the most common causes of cancer death worldwide^13^. Using cancer therapies has its advantages and limitations,as , such as their effect on healthy and cancer cells leading to long-term side effects. Moreover, some cancer treatments depend mostly on blood circulation; therefore, they are less effective in poorly vascularized tumors. The localized therapies reduce the mass of tumors and, in some cases, can cure cancer; however, they cannot treat metastasized cancers^14^.

Therefore, new anticancer drugs with more efficacy and the ability to minimize side effects are needed. In the entire history, natural products demonstrated a dominant role in the treatment of various diseases. For cancer treatment, many studies evaluated the effect of the natural agents to inhibit, retard, or reverse the process of carcinogenesis. Nowadays, natural products represent about 60% of all cancer drugs^15^. Fungi have been shown to produce several important therapeutic products such as penicillin, cyclosporin, statins; mycotoxins such as aflatoxins and trichothecenes, and some anticancer potentials^3,16–20^. Given that the chemical diversity of natural products is based on biological and geographical diversity, researchers are exploring the entire globe for bioprospecting. Hence, the current study is anticipated to impact discovering fungal isolates from desert environments as producers of valuable and novel metabolic compounds. So far, the number of mycological studies on Saudi Arabia's desert soil is rather limited^21–24^. Therefore, this study was undertaken to identify fungi from desert soils in Saudi Arabia and test their antimicrobial and anticancer activities, as well as to explore their molecular and chemical constituents. The current investigation is considered the first report for antibacterial and cytotoxic effects for fungal strains isolated from AlQasab, Tabuk and Almuzahimiyah region of Saudi Arabia.

## Materials and methods

### Microbial strains and cancer cell lines

Microbial strains used in this study were, Gram-positive bacteria (*Staphylococcus aureus* 29,213 and *Enterococcus faecalis* 29,212) and Gram-negative bacteria (*Klebsiella pneumonia* 700,603; and *Escherichia coli* 25,922) obtained from the Culture Collection of laboratory, Riyadh, Saudi Arabia.

Furthermore, for the anticancer biological essay a panel of cell lines were tested including, human breast carcinoma cells MCF-7, MDA-MB-231and KAIMRC1^25^; colorectal adenocarcinoma cells HCT8 and HCT116. All the cell lines were purchased from ATCC, USA, except KAIMRC1, which was isolated and established in the core laboratory facility KAIMRC, Riyadh KSA. All the cancer cell lines related work was performed at King Abdullah International Medical Research Center (KAIMRC) Riyadh, Saudi Arabia.

### Isolation of fungi from desert soil

The soil samples were collected in clean plastic bags, at a depth of 5–20 cm, from different sites of Saudi Arabia's deserts^24^. Fungi were isolated from the soil samples by the dilution method and soil plate method 1,2 using Potato Dextrose Agar (PDA) containing Sabouraud dextrose agar, Czapek-Dox Agar with chloramphenicol 1%. The plates were incubated at 28 °C were observed for one week. After one week, each plate was examined again to obtain purified fungi cultures using serial inoculation. The fungal morphology was studied macroscopically by observing the colony features (color, shape, size, and hyphae) to identify each fungi's genus.

### Molecular identification of fungi

In this study, three isolates were selected and were previously identified in Alotaibi et al. ^24^.

### DNA extraction of fungal strains

DNA from four fungi isolates was extracted using the InstaGene Matrix Genomic DNA Kit (BIO-RAD Laboratories, Hercules, CA, USA) as described in the manufacturer’s instructions.

### PCR amplification and purification

PCR amplification of fungal 18S rRNA genes from desert soil samples was carried out using genomic DNA as the template and a couple of primers, NS1 F (5′ GTAGTCATATGCTTGTCTC 3′) and NS8 R (5′ TCCGCAGGTTCACCTACGGA 3′). Amplification was achieved with the following PCR reaction mixture: 10X Taq PCR Buffer, 2 µl; 2.5 mM dNTP mixture, 1.6 µl; F and R primers (10 pmol/µl), 1.0 µl; KOMA Taq (2.5 U/ µl), 0.2 µl; DNA template (20 ng/µl), 2 µl; and HPLC-grade distilled water to adjust the reaction volume to 20 µl. All PCR samples were carried out using a thermal cycler by initially denatured at 95 °C for 5 min; then amplified by using 30 cycles of 95 °C for 0.5 min, annealing at 55 °C for 2 min and extension at 68 °C for 1.5 min; and a final extension at 68 °C for 10 min. Then, PCR amplification products were separated by 1% agarose gel electrophoresis. The PCR products were purified using the Montage PCR Cleanup Kit (MILLIPORESIGMA, Burlington, MA, USA).

### Sequencing the amplified DNA

The 18S rRNA gene in the purified products was sequenced using the identical primers used for amplification and the BigDyeTerminator v3.1 Cycle Sequencing Kit (THERMO FISHER Scientific, USA). PCR templets were sequenced using 3730xl DNA Analyzer automated DNA sequencing system (THERMO FISHER Scientific, USA) at MACROGEN, Inc. (South Korea).

### Sequence analysis

The sequences of all fungal isolates were edited using Geneious prime software, Geneious Prime Version 2020.1.2^26^. Consensus sequences were generated from forward and reverse sequences. Then, sequences were compared with those of their closely related reference strains using the nBLAST database from the National Center of Biotechnology Information (NCBI) website. Phylogenetic tree constructed using the Neighbor-Joining method in MEGA X [4]^27^.

### Data availability

The 18S rDNA nucleotide sequences of all isolates were deposited at GenBank.

### Screening of biological activity

#### Antimicrobial assay

The antibacterial investigation was done for the fungi isolates against tested bacteria using agar disk diffusion assay^28^. Each fungal isolate was cultured on Sabouraud dextrose agar with chloramphenicol 1% plate, for seven days, at 28 °C. Then, disks (15 mm diameter) were cut from the Sabouraud dextrose agar and placed to the top of different media plate that previously inoculated with bacteria: [Blood agar, (BA)] and [Nutrient agar,(NA)] and [Sabouraud dextrose agar (SDA)]. The plate was incubated at 37 °C for seven days. Antimicrobial activity was evaluated by visualization followed by the measurement of inhibition zones (in mm). The distance across the inhibition zones were grouped as follows: 90–70 mm (^+++^, strong inhibition), 69 to 40 mm (^++^, moderate inhibition), 39–20 mm (^+^, weak inhibition), and less than 15 mm (^−^, no activity).

#### Fermentation and extraction of fungal biomolecules

**Figure 1:**
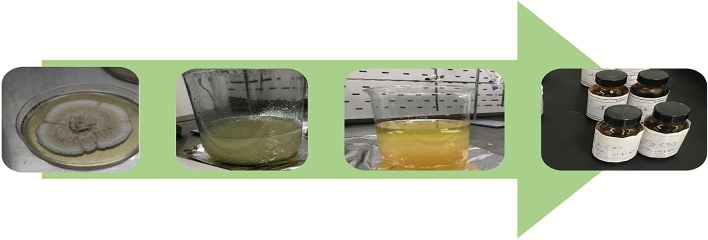
Images show the process of fungal extracts.

The fungi were cultured on PDA and incubated for two weeks at 25° C and then placed at 4 °C for a month. The content of seven plates (9 cm) was transferred to a 2 L beaker. 315 ml brine solution (A fully concentrated sodium chloride solution) was added, and the mixture was homogenized for about 3–5 min using a hand blender. The homogenized mixture was transferred to a large beaker, and 945 ml ethyl acetate was added. Then the mixture was blended for about 10–20 min by hand blender. After that, the formation of two layers (aqueous and organic layers). Each layer was drawn into a 1000 ml conical flask. The solvent was removed under reduced pressure in a rotary evaporator. The extracts were collected, and their weights were recorded then kept at 4 °C (Fig. 1).

#### Analysis of the fungal extract by analytical RP-HPLC method

The above obtained extracts were subjected to HPLC–DAD followed by LC/Ms. Chemicals of formic acid and HPLC grade Methanol were purchased from SIGMA-ALDRICH (St. Louis, MO, USA) and Honeywell (France), respectively. The three fungal extracts were first injected into Agilent1260 Infinity HPLC system (AGILENT, Germany) with Diode-Array Detection (DAD) detector. The separation carried out in a reverse phase mode using Phenomenex Kinetex-C18 column (4.6 mm × 250 mm, 5 μm) with the following elution gradient; 0–1 min, 5% B; 1–11 min, 5–100% B; 11–13 min, 95%B; 13–15 min, 5%B; 15–16 min, 5%B using mobile phase A (0.1% HCOOH in water) and mobile phase B (0.1% HCOOH in Methanol). Samples were injected with 20 and the flow rate was set as 1 ml/min. The DAD collected UV spectrum at 200, 225, 250, 275, 300, 325, and 350. The data was processed using Chemstation software.

### LC–QTOF-MS method

The analysis of all extracts was performed on Agilent1260 Infinity HPLC system (AGILENT, Germany) coupled to Agilent 6530 Quadrupole Time of Flight (AGILENT, Singapore). Separation was performed using Agilent Extend-C18 column (2.1 mm × 50 mm, 1.8 μm) with the following elution gradient; 0–1 min, 5% B; 1–11 min, 5–100% B; 11–13 min,95%B; 13–15 min, 5%B; 15–16 min, 5%B using mobile phase A (0.1% HCOOH in water) and mobile phase B (0.1% HCOOH in Methanol). The injection volume was 10 µl, and the flow rate was set as 300 µl/min. The MS1 acquisition method was directly utilized for acquisition of positive-ion and negative-ion Electrospray Ionization (ESI) mass spectra mode. The cone voltage was adjusted in the range between 25 and 50 V. Fifty scans from 100 to 600 Daltons were collected to generated the averaged spectra*.* The capillary voltage was optimized at 4.5 kV in positive ion mode and − 3.1 kV in negative ion mode. The mass spectrometer parameters were set as follows: Gas Temperature = 300 °C; Gas flow = 8 I/min; Nebulizer = 35 psig; Sheath Gas Temperature = 350 and Sheath Gas flow was 11. MS1 data was generated by Agilent Mass Hunter qualitative analysis software.

### Cell viability assay

The CellTiter-Glo assay (Promega) was used to evaluate the cytotoxicity of fungal extracts which were extracted at the PNU including *Chaetomium* sp., *F. venenatum*, and *Bipolaris* sp. against the MCF-7, MDA-MB-231, and KAIMRC1 (breast cancer cell lines) and HCT8 and HCT116 (colorectal cancer cell lines). Cancer cells were plated on flat-bottom white 96-well plates at a density of 5 × 10^3^ cells/well in 100 μL growth medium. Serial dilutions of the extracts, ranging from 100 to 0.01 μg/100µL cell culture media, were made in triplicates and transferred to the cell culture plates containing the cells. Additional rows with only cells were added to account for the compounds and cells' effect. Cells were incubated for 48 h at 37 °C with 5% CO_2_. Cell viability was determined using the CellTiter-Glo assay according to the manufacturer’s recommendations. Luminescence was measured using the Envision plate reader (PERKIN ELMER). Luminescence readings were normalized to averaged DMSO controls and expressed as a relative percentage. Mitoxantrone was used as a positive control. IC_50_ (µg/ml) less than 10 µg/ml is considered strongly active; 11–100 µg/ml is considered moderately active, and above 100 µg/ml is considered not active.

### Statistical analysis

Quantitative variables were performed using GRAPHPAD PRISM 8.1 software for the half-maximal inhibitory concentration (IC_50_) graphs. Concentrations were transformed to log10.

Then the data were normalized, 0–100%, 0% is always the first point that is the highest concentration of the compound, and 100% is DMSO control (no killing). On the plot, error bars denote standard deviation (SD), and R squared (coefficient of differentiation) values for each compound represents the proportion of variance for a variable. All the measurements were repeated thrice.

## Results

### Isolation of the fungi

**Figure 2:**
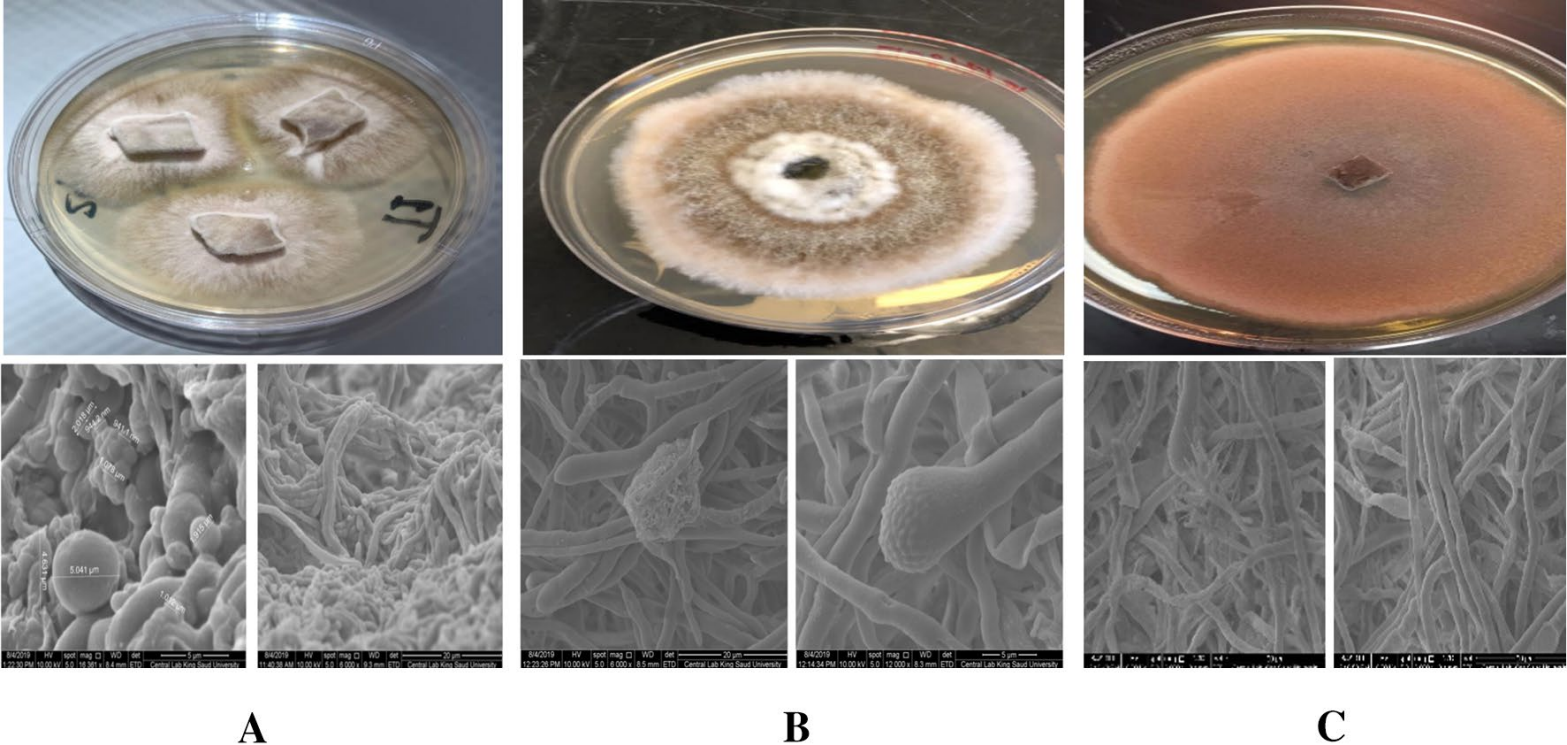
The colony characteristics in PDA plates and under SEM of the three endophytic fungi *Chaetomium sp* (**A**), *Bipolaris* sp. (**B**) and *Fusarium venenatum* (**C**) isolated from Tabuk, AlQasab and Almuzahimiyah, respectively.

In this study, three culturable fungal isolates were collected from different desert soils in Saudi Arabia, including *Cochliobolus sp.* (*Bipolaris sp.*) and *Chaetomium* sp., were from AlQasab and Tabuk, respectively^24^, and *F. venenatum* was isolated from Almuzahimiyah. The isolates have been recognized in relation to colonies and conidia following the appearance of* colonies* on PDA medium after five days (Fig. 2). White with grayish dark brown color, a dry, flocculent, and fluffy appearance was noted for *Chaetomium* sp., a mixture of black and white colonies was noticed for *Bipolaris* sp. however, the black colonies and pigments are considered as a sign for fungal ability in melanin production. Furthermore, the growth of *F. venenatum* in PDA plate colonies with a distinctive rose-red pigmentation was noted. Furthermore, mycelia were branched (Fig. 2).

Moreover, the molecular characteristics of isolated strains were performed based on 18S DNA for more fungal identification.

### Molecular characterization of the fungal isolates

**Figure 3:**
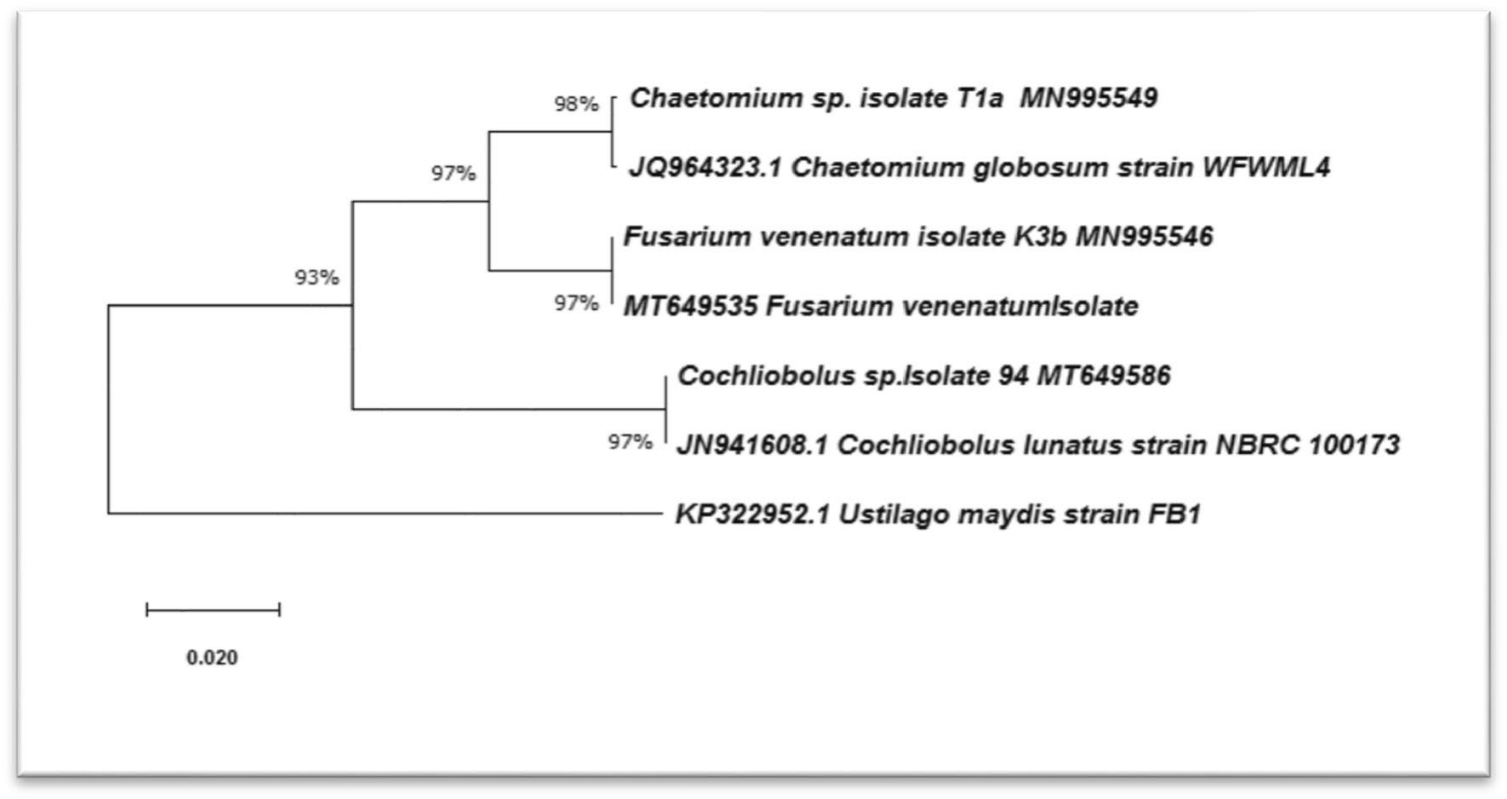
Phylogenetic tree of fungi isolates based on 18S ribosomal DNA.

The 18S rDNA sequences of the isolated fungal strains were compared to the GenBank sequence database information for genera or species identification using BLASTn. The presence of three fungal genera presented 100% similarity to *Chaetomium* sp. (MN995549)*, Bipolaris* sp. (MT649586), and *F. venenatum* (MT649535). For phylogenetic tree construction, the homology sequence from the BLASTn analysis was noted and showed the relationships among various fungi species isolated from soil (Fig. 3). Phylogenetic analysis indicated that the isolates used in this study and similar species in the GenBank.

Phylogenetic tree constructed using the Neighbor-Joining method in MEGA X[4]^27^. Bootstrap values were generated from 1000 replicates. This analysis involved three nucleotide sequences, the accession number shown at the end of the branch and their closest relatives’ sequences from the GenBank accession number shown at the tip of the branch. *Ustilago maydis* accession number KP322952 was as an outlier. The percentage of replicate trees in which the associated taxa clustered together are shown next to the branches.

### Antimicrobial activity screening of identified fungi

The three isolated fungal strains: *Chaetomium* sp., *Bipolaris* sp., and *F. venenatum* were assessed for antibacterial effect against two Gram-positive *S. aureus* and *E. faecalis,* as well as two Gram-negative bacteria; *E. coli* and *K. pneumonia* using different growth media. The tested isolates demonstrated various levels of inhibitory activity (from low to high selected)^29,30^ against tested bacterial strains (Table 1). A varied range of inhibitory activity was noted for the different fungal isolates used in terms of fungal type, microbial strain tested, and growth condition.

**Table 1:** Antimicrobial activity screening of fungi from desert soil.

Growth condition	Fungal isolates	Gram-positive		Gram-negative	
		*S. aureus*	*E. faecalis*	*E. coli*	*K. pneumonia*
Blood agar	*Chaetomium* sp.	20^+^	NE*	NA	NE*
	*F. venenatum*	55^++^	40^++^	65^++^	50^++^
	*Bipolaris* sp.	NA	20^+^	20 ^+^	NA
Sabouraud dextrose agar	*Chaetomium* sp.	41^++^	NA	27 ^+^	20 ^+ ^
	*F. venenatum*	80 ^+++^	84^+++^	80 ^+++^	50^++^
	*Bipolaris* sp.	73^+++^	65^++^	78^+++^	30^+^
Nutrient agar	*Chaetomium* sp.	45^++^	20^+^	65^++^	25^+^
	*F. venenatum*	85^+++^	80^+++^	80^+++^	85 ^+++^
	*Bipolaris* sp.	80^+++^	38^+^	84^+++^	75^+++^

Inhibition Zone, 90–70 mm (^+++^, strong inhibition), 69–40 mm (^++^, moderate inhibition), 39 –20 mm (^+^, weak inhibition), and less than 15 mm (NA) (−, no activity) and *No effect (NE).

It was also noted that, in blood agar, the inhibitory activity for *F. venenatum* and *Bipolaris* sp. were higher against the four tested bacteria compared to *Chaetomium* sp. (Table 1) where *E. coli* was the most affected microbe. However, *Chaetomium* sp. had antimicrobial activity only against *E. coli and S. aureus*. Furthermore, when the fungal isolates were tested in sabouraud dextrose agar, activities against all pathogenic bacteria were noted, however, the activity of *Chaetomium* sp. was lower against all tested microbes compared to that of *F. venenatum* and *Bipolaris* sp. (Table 1). On the other hand, for the fungal isolates tested in nutrient agar, the efficiency against all pathogenic bacteria was noticed. The activity of *Chaetomium* sp. was also lower against all tested microbes compared to the activity of *F. venenatum* and *Bipolaris* sp. (Table 1). No noteworthy trend of observation was observed for the funagl activity against tested microbes in all conditions; however, *Bipolaris* sp. showed higher activity against Gram-negative compared to Gram-positive bacteria when tested in nutrient agar.

### Cytotoxicity assay

**Figure 4:**
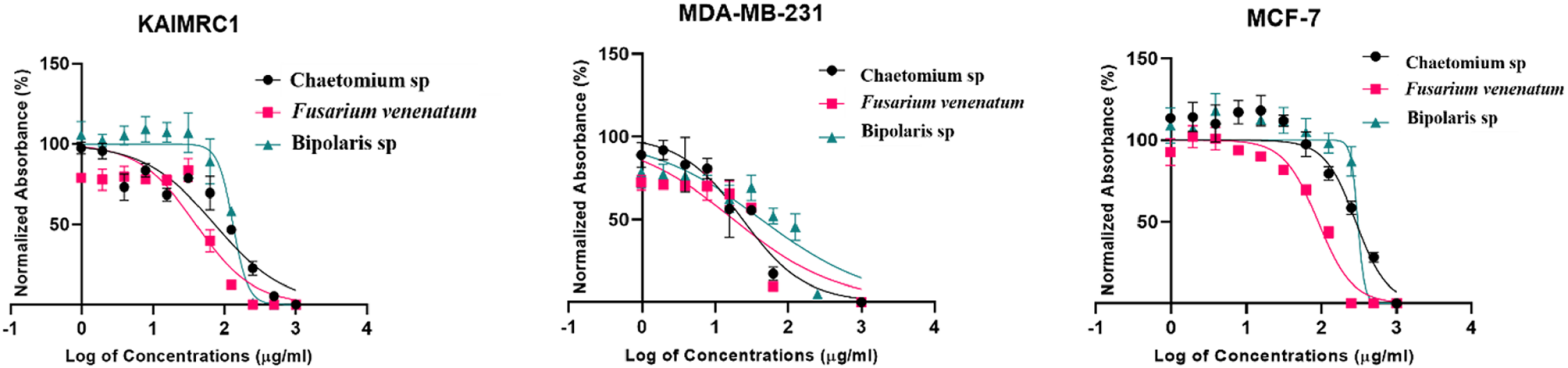
Dose–response manner and the inhibition curve of the three tested crude extracts on different breast carcinoma cells.

**Figure 5:**
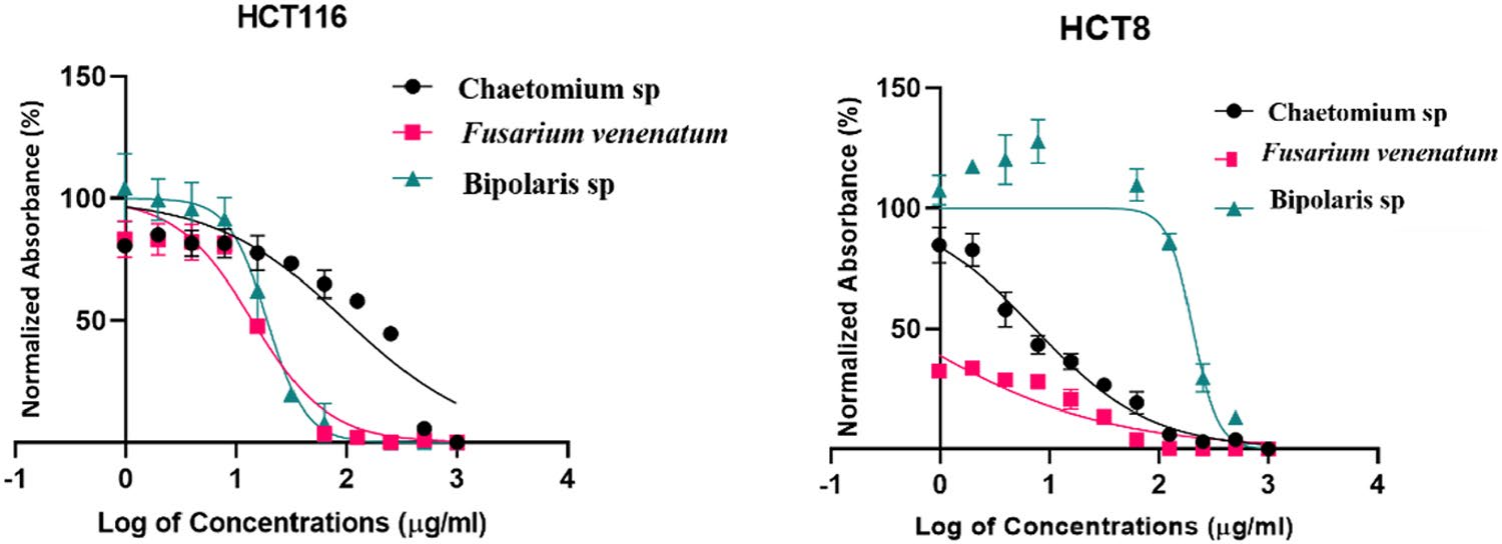
Dose–response manner and the inhibition curve of the three tested crude extracts on different colorectal carcinoma cells.

The anticancer assay was an attempt to determine the effect of the fungal extracts on cell proliferation. To achieve this goal MCF-7, MDA-MB-231, KAIMRC1 (breast cancer cell lines), HCT8 and HCT116 (colorectal cancer cell lines) were utilized. The half-maximal inhibitory concentration IC_50_ (µg/ml) of each fungal extract (Table 2) was stated by plotting a dose–response curve (Figs. 4, 5) and discussed according to the preliminary screening assays by American National Cancer Institute guidelines (NIC). In our study, we refer that the crude extracts attaining 50% cytotoxic effect at a concentration < 10 μg/ml is considered as strongly active and that between 11–100 μg/ml is considered moderately active and that above 100 µg/ml is considered as non-active^31,32^.

**Table 2:** The half-maximal inhibitory concentrations (IC_50_) of fungal extracts for the five human cancer cell lines.

Treatments	IC50 (µg/ml)				
	Breast cancer cell lines			Colorectal cancer cell lines	
	MCF7	MDA- MB-231	KAIMRC1	HCT8	HCT116
*Chaetomium* sp.	335.6	23.61	75.12	8.744	152.8
*F. venenatum*	90.44	12.52	37.69	0.3779	15.86
*Bipolaris* sp.	301.9	39.93	142.0	202.5	18.97
Mitoxantrone	1.2	0.52	0.65	0.32	0.7

### Cytotoxicity against breast cancer cell lines

Three breast cancer cell lines (MCF-7, MDA-MB-231, KAIMRC1) were tested under three crude fungal extracts, and Mitoxantrone as a positive control. The effect of the crude extracts from the investigated fungal strains was statistically different at P ≤ 0.05 level, although in some cases, the antiproliferative activity was low. The crude extract from *F. venenatum* showed moderate antiproliferative activity against all breast cancer cell lines. A better noticed activity was against MDA-MB-231since lower IC_50_ was noted (12.52 µg/ml) compared to IC_50_ for other breast cancer cell lines. Furthermore, extract from *Chaetomium* sp. had moderate activity against MDA-MB-231and KAIMRC1 and showed IC_50_ 23.61 and 75.12 µg/ml, respectively; however, no activity was noted against MCF-7 (IC_50_ 335.6 µg/ml) as indicated in Fig. 4. Furthermore, the crude extract from *Bipolaris* sp. isolate showed moderate antiproliferative activity against MDA-MB-231(IC_50_ 39.93 µg/ml); however, no activity was noted against KAIMRC1 and MCF-7. On the other hand, the MDA-MB-231cell lines were very sensitive to all crude fungal extracts; however, KAIMRC1 cells were sensitive to crude extract of *Chaetomium* sp. and *F. venenatum* and MCF-7 cells were only sensitive to *F. venenatum*.

### Cytotoxicity against colorectal cancer cell lines

Colorectal cancer cell lines (HCT8 and HCT116) were also tested under the three fungal crude extracts. The results of crude extract from *F. venenatum* showed potent antiproliferative activity against HCT8 cells. We recorded the lowest IC_50_ of 0.3779 µg/ml, comparable to the IC_50_ of the positive control Mitoxantrone (0.32 µg/ml) as presented in Fig. 5. Moderate antiproliferative activity was also noted against HCT116. On the other hand, crude extract from *Chaetomium* sp. showed strong activity against HCT8 (IC_50_ 8.7 µg/ml), and *Bipolaris* sp. showed moderate antiproliferative activity against HCT116 cell lines (18.97 µg/ml).

In brief, the cell line HCT8 was sensitive to *Chaetomium* sp. and *F. venenatum* crude extracts; however, the HCT116 cell line was sensitive to *Bipolaris* sp. and *F. venenatum* crude extracts. The antiproliferative activity was generally noticed in a dose-dependent style for all crude extracts tested against the different cell lines (Fig. 4). On the other hand, the result for each extract was compared to the anticancer activity of Mitoxantrone, which was used as a positive control and showed a remarkable effect on all breast and colorectal cancer cell lines.

**Figure 6:**
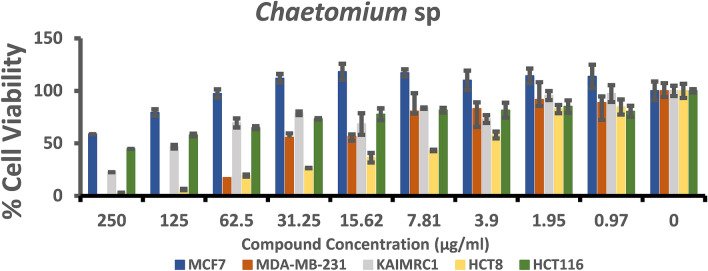
A summary of the biological activity of *Chaetomium* sp extracts on MCF7, MDA-MB-231 and KAIMARC1 breast carcinoma cells as well as HCT8 and HCT116 colorectal carcinoma cells after 96 h of exposure. Columns in the histograms represent the mean ± SD (n = 3) of fungal crude extracts tested at nine different concentrations ranging from 0.97 to 250 μg/ml. The columns represent variations among various concentrations of extracts.

**Figure 7:**
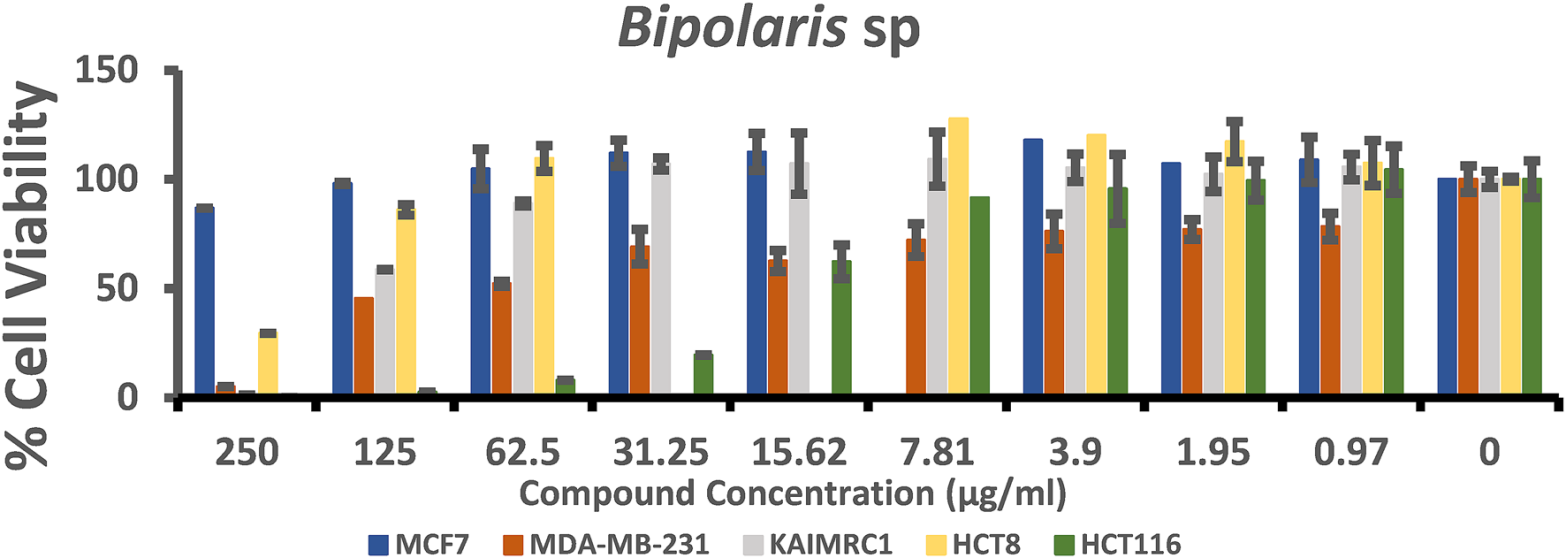
A summary of the biological activity of *Bipolaris* sp extracts on MCF7, MDA-MB-231 and KAIMARC1 breast carcinoma cells as well as HCT8 and HCT116 colorectal carcinoma cells after 96 h of exposure. Columns in the histograms represent the mean ± SD (n = 3) of fungal crude extracts tested at nine different concentrations ranging from 0.97 to 250 μg/ml. The columns represent variations among various concentrations of extracts.

**Figure 8:**
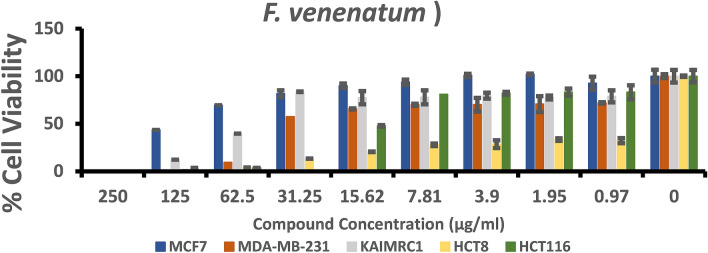
A summary of the biological activity of *Fusarium venenatum* extracts on MCF7, MDA-MB-231 and KAIMARC1 breast carcinoma cells as well as HCT8 and HCT116 colorectal carcinoma cells after 96 h of exposure. Columns in the histograms represent the mean ± SD (n = 3) of fungal crude extracts tested at nine different concentrations ranging from 0.97 to 250 μg/ml. The columns represent variations among various concentrations of extracts.

Furthermore, the three fungal extracts showed biological activities against tested cell lines summarized in Figs. 6, 7, and 8 when nine different concentrations (µg/ml) were applied.

**Figure 9:**
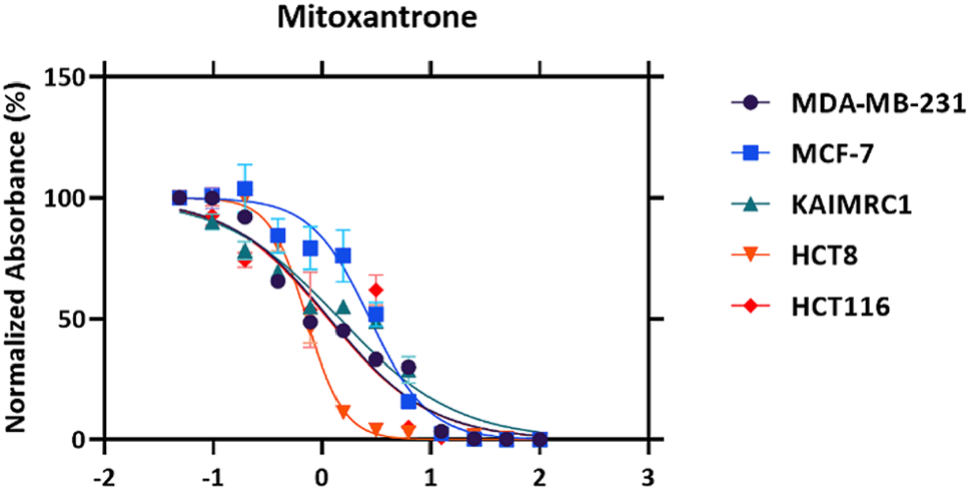
The dose–response manner and the inhibition curve of the positive control (Mitoxantrone ) on different breast and colorectal carcinoma cells.

Furthermore, the dose response curve for the Mitoxantrone against all tested cell lines is presented in Fig. 9.

### Metabolite profiling of fungal extract by HPLC–DAD coupled to analytical LC–QTOF-MS

**Figure 10:**
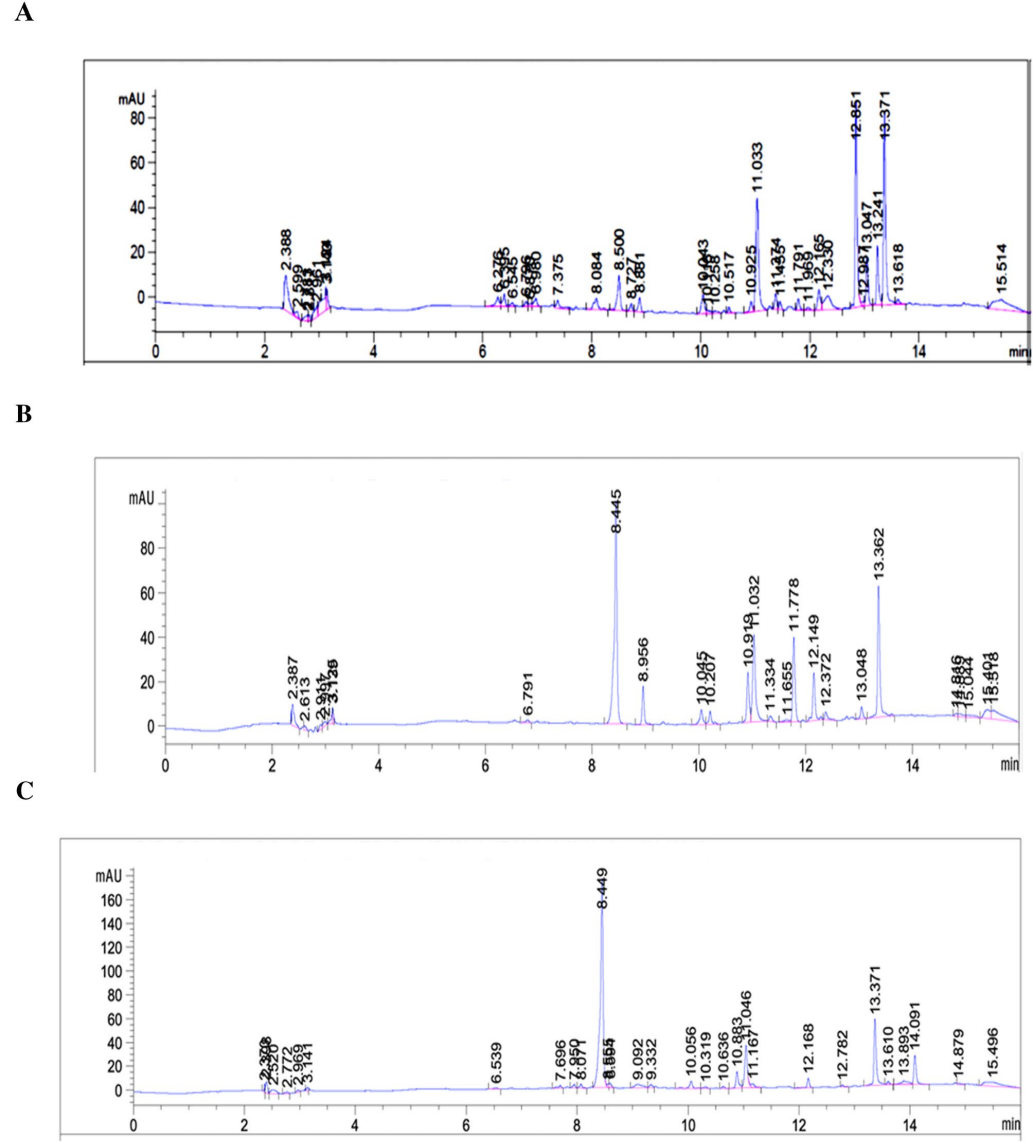
Chemical analysis of the fungal extracts, *Chaetomium* sp, (**A**), *F. venenatum* (**B**) and *Bipolaris* sp. (**C**) by Analytical HPLC–DAD Chromatograms at 275 nm.

Up to date, knowledge about bioactive ingredients from soil fungi is limited. Therefore, to improve our understanding of the potential role of soil fungi, the profile of secondary metabolites of the *Chaetomium sp, Bipolaris* sp. and *F. venenatum* ethyl acetate crude extracts were identified. The amount of the extract’s weights subjected to HPLC–DAD Chromatograms analysis were 0.89, 1.45, and 1.05 g from *Chaetomium sp, Bipolaris* sp. and *F. venenatum*, respectively. An untargeted screening approach initially detected secondary metabolites. Secondary metabolites have been spotted by the information from spectra and molecular weight, matched with reference compounds from the online databases. The fungal extracts' chemical analysis by Analytical HPLC–DAD Chromatograms was done at a wavelength 275 nm and presented in Fig. 10 for the three fungal extracts. Furthermore, the HPLC chromatograms revealed that majority of the peaks appeared at the area of lipophilic range with acetonitrile from 50 to 90%. These peaks retention time and mass values were scanned with the literature reviews and presented for each extract.

### Chemical analysis using LC/MS of the fungal extracts by analytical LC/MS-QTOF

For the analysis of the total ion current spectra (TIC) raw data, the data-analysis program Mass Hunter (Agilent Technologies) qualitative analysis software has been used. Chemical features were extracted from the LC–MS data using the Molecular Features Extraction (MFE) algorithm and the recursive analysis workflow. Features have been extracted by screening the detected nodes at various retention time per minutes, with a minimum intensity of 6,000 counts and aligned with previously detected compounds considering adducts ([M + H]^+^, [M + Na]^+^, [M + K]^+^).

#### *Chaetomium globosum* extract

**Figure 11:**
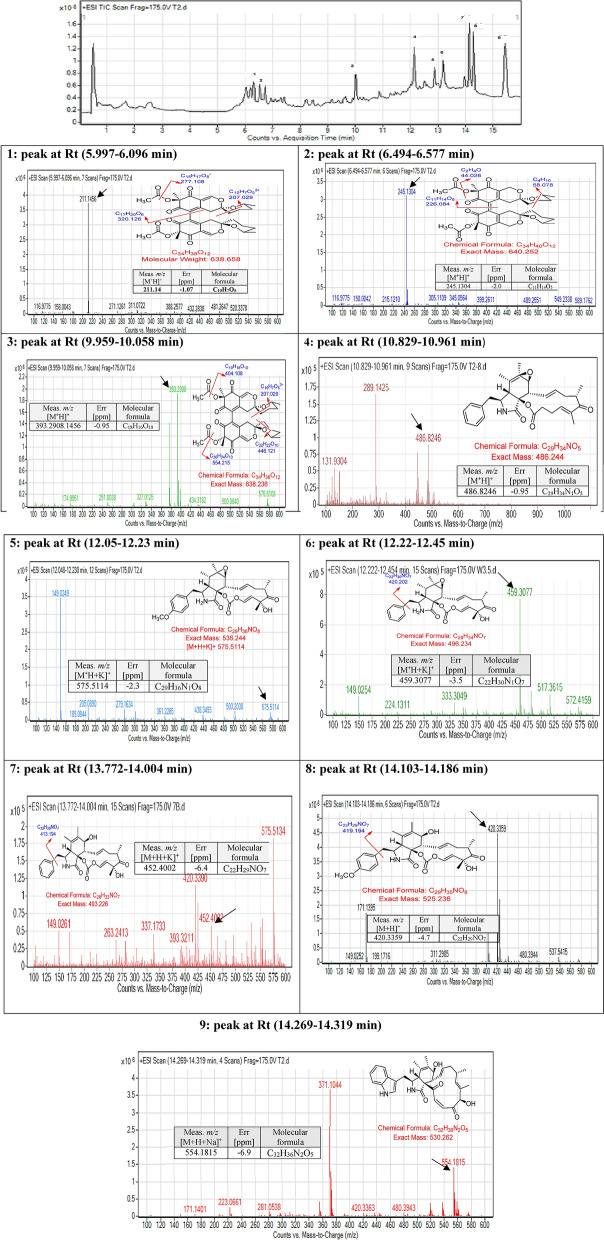
Base peak chromatogram of *Chaetomium globosum* crude extract and identified secondary metabolites which are: Cochliodone A (1), Cochliodone J (2), Cochliodone A (3), Rosellichalasin [2] (4), Cytochalasin B (5), Cytochalasin E (6), Cytochalasin K (7), Scoparasin A (8), Chaetoglobosin (9). Meas m/z implies measured m/z.

Nine major symmetrical peaks were detected in HPLC at a wavelength of 275 mAU for *Chaetomium globosum* extract, and their mass values of the major peaks vary from 100–600 daltons *m/z*. Those mass values were found to match specific certain compounds isolated previously. The mass spectroscopy of *Chaetomium globosum* extract is shown in Fig. 11.

A mass screening on the above spectrum was conducted and summarized as follows, the screened area from 6–7 min showed specific mass value peaks, two peaks were detected and studied. While seven peaks were detected for the peaks from 10–16 min, studied and analyzed extensively. Generally, detected compounds showed varied mass values ranged between 200–600 daltons *m/z*. Detected mass values were correlated with the previously identified compounds. They provided nine compounds including, Cochliodone A, Cochliodone J, Cochliodone A, Rosellichalasin [2], Cytochalasin B, Cytochalasin E, Cytochalasin K, Scoparasin A, Chaetoglobosin^33,34^ as presented in Fig. 11.

For the first peak detected at retention time (Rt) (5.997–6.096 min), the parent compound is Cochliodone A^33^ with *m/z* 638.658 daltons and a molecular formula of [C_34_H_38_O_12_]^+^ that correlated with a fragmented product of *m/z* 211.1456 daltons in positive ion mode and [M-H]^-^ with *m/z* 210 daltons in negative mode, indicating that the compound have a molecular weight of 211 g mol^−1^ and molecular formula [C_10_H_7_O_5_]^+^. The second peak at Rt (6.494–6.577 min) represented the parent compound Cochliodone J^33^ with *m/z* 640.252 daltons and a molecular formula [C_34_H_40_O_12_]^+^. The second peak was correlated with a fragmented product of *m/z* 245.1304 daltons [C_11_H_14_O_5_]^+^ in positive ion mode and [M-H]^-^ with *m/z* 244 daltons in negative mode, indicating that the compound have a molecular weight of 245 g mol^-1^ [C_11_H_14_O_5_] ^+^. The third peak at Rt (9.959–10.058 min) showed the parent compound Cochliodone A^33^ with *m/z* 638.658 daltons in positive ion mode and [M-H]^-^ with *m/z* 637 daltons in negative mode, indicating that the compound have a molecular weight of 638 g mol^-1^ and a molecular formula of [C_34_H_38_O_12_]^+^. The third peak was correlated with a fragmented product of *m/z* 393.059 daltons [C_18_H_16_O_10_]^+^. The fourth peak at Rt (10.829–10.961 min) was correlated with the compound Rosellichalasin [2]^34^ with *m/z* 486.8246 daltons in positive ion mode and [M-H]^−^ with *m/z* 485 daltons in negative mode, indicating that the compound have a molecular weight of 486 g mol^-1^and a molecular formula of [C_29_H_34_NO_5_]^+^. The fifth peak at Rt (12.05–12.23 min) was correlated and identified as Cytochalasin B cited previously by Shen Y. et al.^34^. The identified compound with *m/z* 536.244 daltons is the compound with potassium since the experiment were run in a positive mode with the chemical formula [C_29_H_36_N_1_O_8_]^+^. The Electrospray Ionization mass spectra (ESI–MS) of this node which appears at Rt (12.05–12.23) correlated with a potassiated ion peaks *m/z* 575 daltons in positive ion mode and [M-H]^-^ with *m/z* 537 daltons in negative mode, indicating that the compound have a molecular weight of 536 g mol^-1^. For the sixth peak at Rt (12.22–12.45 min), the parent compound is Cytochalasin E^34^ with *m/z* 496.234 daltons and a molecular formula of [C_34_H_40_O_12_]^+^.The ESI–MS of this node which appears at Rt (12.22–12.45 min) correlated with a potassiated ion peaks *m/z* 459 daltons in positive ion mode and [M-H]^-^ with *m/z* 419 daltons in negative mode, indicating that the compound have a molecular weight of 420.2 g mol^-1^. For the seventh peak at Rt (13.772–14.004 min), the parent compound is Cytochalasin K^34^ with *m/z* 493.226 daltons and a molecular formula of [C_28_H_33_NO_7_]^+^. The ESI–MS of this node which appears at Rt (13.772–14.004 min) correlated with a potassiated ion peaks *m/z* 452 daltons in positive ion mode and [M-H]^-^ with *m/z* 412 daltons in negative mode, indicating that the compound have a molecular weight of 413 g mol^-1^. The eighth peak at Rt (14.103–14.186 min), the parent compound is Scoparasin A^33^ with *m/z* 525.236 daltons and a molecular formula [C_29_H_35_NO_8_]^+^. The peak that appeared at Rt (14.103–14.186 min) is correlated with a fragmented product of *m/z* 419.194 daltons in positive ion mode and [M-H]^-^ and molecular formula [C_22_H_29_NO_7_] ^+^. The final peak at Rt (14.269–14.319 min) is correlated and identified to be Chaetoglobosin B, which is cited previously^34^. The identified compound with *m/z* 554 daltons and molecular formula of [C_32_H_36_N_2_O_5_]^+^. The ESI–MS of this node which appears at Rt (14.269–14.319 min) correlated with a sodiated ion peaks *m/z* 554 daltons in positive ion mode and [M-H]^-^ with *m/z* 529 daltons in negative mode, indicating that the compound have a molecular weight of 530 g mol^-1^.All peaks and related chemical compounds are presented in Fig. 11.

#### *Fusarium venenatum* extract

**Figure 12:**
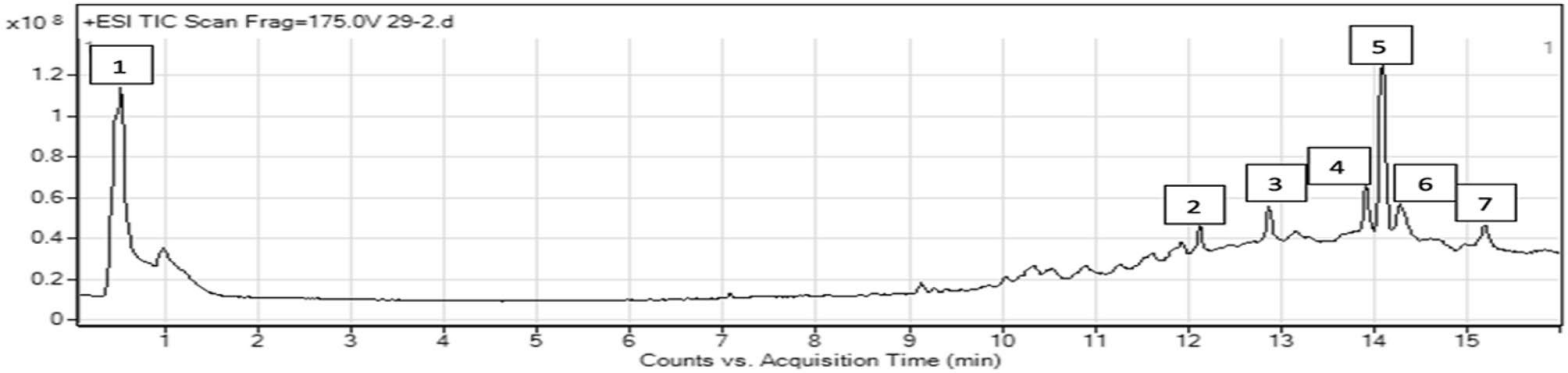
Metabolomic profiling using LC–MS, chromatogram of* F. venenatum* extract presenting the molecular mass for different compounds.

Several studies investigated the extract of *F. venenatum* and confirmed its bioactivity (Fig. 12); however, no metabolites have been previously identified. Therefore, no information is available for comparison with detected peaks in our study (Supplmentary file).

#### *Bipolaris* sp. extract

**Figure 13:**
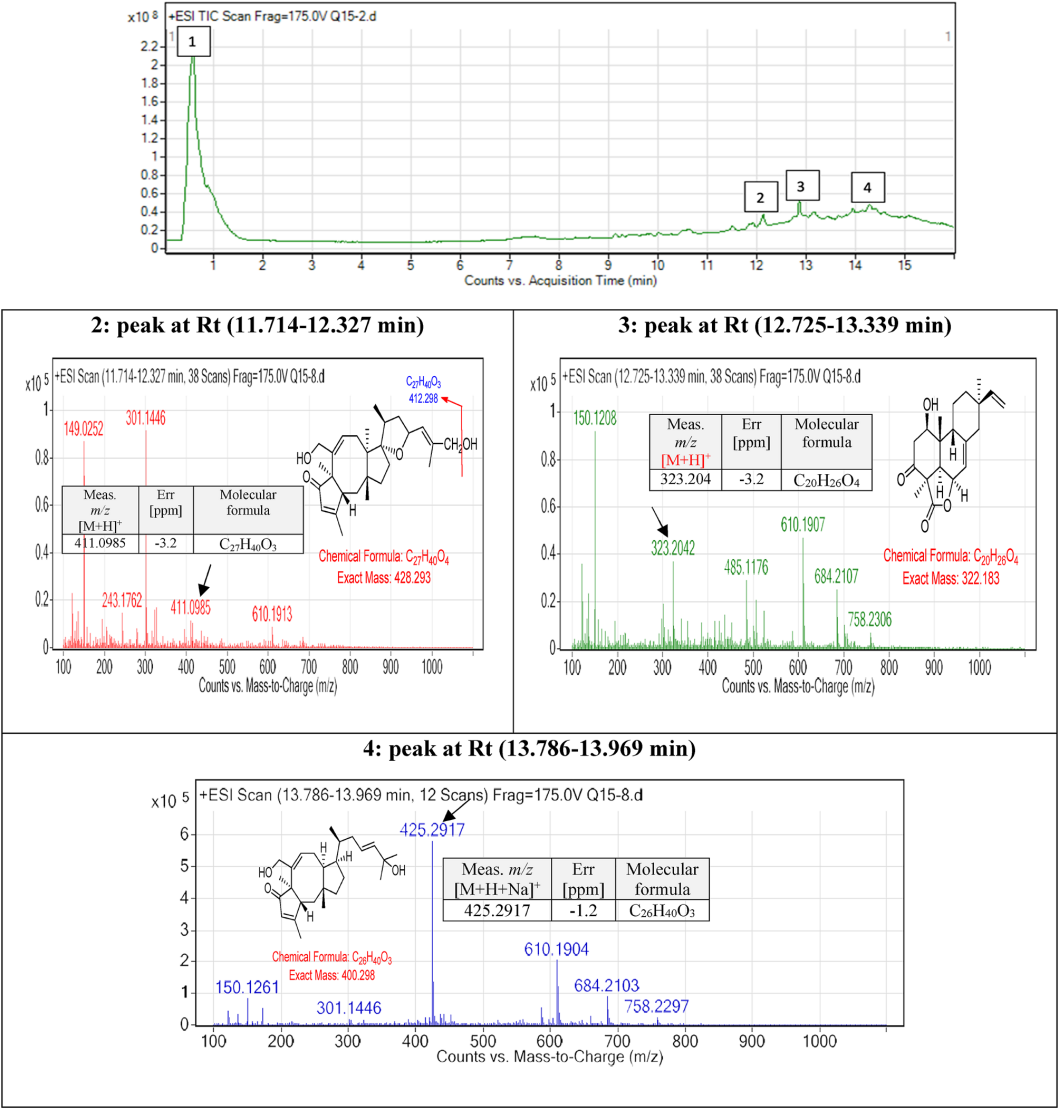
Metabolomic profiling using LC–MS, chromatogram of *Bipolaris* sp. extract presenting the molecular mass different compounds and base peak chromatogram of *Bipolaris* sp. crude extract and identified secondary metabolites which are: (**A**) is 25-hydroxyphiobolin I, (**B**) is1β-hydroxy momilactone and (**C**) is bipolatoxin E. Meas m/z implies measured m/z.

Spectrum for *Bipolaris* sp. extract was screened, searching for the previously identified compounds. The mass screening was conducted, and three specific mass value peaks were detected and studied. From 11–16 min, peaks were studied and analyzed extensively (Fig. 13). Generally, the detected compounds showed varied mass values ranged from 300–500 daltons *m/z*. By correlating this mass value with the previously identified compounds^35^ the following conclusions were driven (Fig. 13).

The peak at Rt (11.714–12.327 min) showed the parent compound 25-hydroxyphiobolin I^35^ with *m/z* 428.293 daltons and a molecular formula of [C_27_H_40_O_4_]^+^. The peak is correlated with a fragmented product of *m/z* 411.0985 daltons [M + H]^+^ and molecular formula [C_27_H_40_O_3_]^+^. Furthermore, at Rt (12.725–13.339 min) peak is correlated and identified to be 1*β*-hydroxy momilactone A, which is cited previously by Shen. et al.^35^. The identified compound had *m/z* 322.183 daltons and chemical formula [C_20_H_26_O_4_]^+^. Furthermore, the peak at Rt (13.786–13.969 min) was correlated and identified as bipolatoxin E, which is mentioned previously by Shen. et al.^34^ with *m/z* 425 daltons and molecular formula of [C_26_H_40_O_3_]^+^. The ESI–MS of this node which appears at Rt (13.786–13.969 min) correlated with a sodiated ion peaks *m/z* 425 daltons in positive ion mode and [M-H]^-^ with *m/z* 401 daltons in negative mode, indicating that the compound have a molecular weight of 400 g mol^-1^.

## Discussion

The application of chemical agents in controlling microbes and cancer is now facing significant challenge due to the increased microbial resistance and their side effects. The increasing microbial resistance by human pathogenic bacteria and fungi to existing drugs has become a global concern^36^. Hence, there is a need to look for other safer and more sustainable approaches in controlling infections.Screening for antimicrobial metabolites from fungi isolated from extreme ecosystems could be an alternative strategy to identify novel drugs. The natural bioactive materials from microorganisms have gained much-needed attention regarding antimicrobial drugs and cancer treatment in the last decade. Therefore, the current investigation partially relayed on some different fungal strains identified from different soils in Saudi Arabia^24^. Currently, some biological activities from such organisms’ inhabitants’ harsh conditions being addressed. The secondary metabolites produced by these microorganisms could be among the main strategies for such microbes to overcome and survive under these extreme conditions. In the present investigation, isolates identified based on colonies that initially appeared white with grayish dark brown color, a dry, flocculent, and fluffy appearance for *Chaetomium* sp. Results were similar to those presented by Yang et al.^37^.

Combination of black pigment and white colonies were noticed for *Bipolaris* sp. Similar findings were also recorded by Tapfuma et al^38^. The ability of the fungal strain in melanin production in hyphae might increase fungal ability in stress tolerance and uptake of nutrients^39^. *F. venenatum* showed pink colonies. Furthermore, based on 18S rDNA sequence analysis, it was revealed that the fungal isolates belong to *Chaetomium globosum*, *F. venenatum*, *Bipolaris* sp. and the phylogenetic analysis showed the similarity between these isolates and those previously deposited in GenBank. Session numbers of MN995549, MT649586 and MT649535 were recorded for *Chaetomium* sp., *Bipolaris* sp. and *F. venenatum,* respectively. The current investigation revealed promising biological activities for the tested fungal extracts; therefore, it was likely necessary to find out the extracts' metabolomic profiling to determine the active components. Such a trial was an attempt to detect some active ingredients from fungal origin that might offer lead compounds for drug discovery.

Our investigation demonstrated a varied range of inhibitory activity for the different fungal isolates tested. Variations in the inhibitory efficiency against the tested bacterial strains could be related to the fungal, microbial strain tested, and growth media. Fungi from soil could be a source for various kinds of antimicrobial compounds^40^. Many studies have reported the discovery of various antimicrobial compounds from fungi such as; peptides, phenols, alkaloids, quinones, flavonoids, steroids, and terpenoids^41,42^. *Fusarium, Aspergillus, Cladosporium, Penicillium*, and *Yeasts* showed the ability to produce metabolic compounds with antimicrobial ability^43,44^.

Regarding *Chaetomium globosum*, the lower inhibitory activity was shown against the tested bacteria at all test media compared with the other two extracts activity. *Chaetomium* NoS3 exhibited antibacterial activity against *S. aureus*, *Salmonella anatum*, *Bacillus cereus*, *Listeria monocytogenes,* and *E. coli* with MIC value ranging between 1 and 10 mg ml^-1^^44^. Ethyl acetate and dichloromethane extracts from *Chaetomium* sp. showed inhibitory effects on *Klebsiella pneumonia*, *Bacillus subtilis*, and *S. aureus*^45^ as well against methicillin-resistant *S. aureus*^46^. A previous study by Ge et al.^47^ revealed that *Chaetoglocins* A and B belong to pyranones compounds extracted from *C. globosum* strain IFB-E036, showed significant antimicrobial activity against the Gram-positive bacteria with minimum inhibitory concentration (MIC) value ranging between 8 and 32 μgml^−1^. However, numerous new secondary metabolites can be extracted from *Chaetomium* species exhibiting many antibiotic effects, including orsellides A–E^48^. Another study by Momesso et al.^49^ revealed a weak inhibition effect from Chaetoglobosin B against two bacterial strains *E. coli* and *S. aureus,* with MIC values of 189 and 120 μgml^−1^, respectively. Significant antibacterial activity of *Chaetosidone* A, *corynesidone* B, and corynether against *S. aureus* and *B. subtilis* at concentrations of 40 μg per disc was noted^50^.

Our results are in accordance with previous studies where acetonitrile extract of mycoprotein from *F. venenatum* showed substantial antimicrobial activity against *Staphylococcus aureus*^51^. Contradictory observation was noted by Sondergaard et al.^52^, who showed the presence of mycelium pigment bikaverin from *F. venenatum* that exhibited antimicrobial effects. However, inhibited *Lactobacillus acidophilu,* but no activity against *E. coli* and *S. aureus* was noted, which might be due to different test conditions. Besides, the fractions compounds from *F. poae* and *F. solani* possess an antibacterial effect against *S. aureus*^52^. Many studies revealed secondary metabolites from *Fusarium* sp. possessing an antibacterial effect, such as antibiotic Y, beauvericin, enniatins, and fusaric acid^53–58^.

On the other hand, *Bipolaris* sp. showed high efficiency as an antimicrobial agent against all test pathogenic bacteria, which is in accordance with Shen et al.^35^, who reported the presence of some antimicrobial compounds from *Bipolaris* species TJ403-B1 such as bipolatoxin D and ophiobolin A. In detail, bipolatoxin D compound exhibited inhibition effects against *Enterococcus faecalis* with a MIC value of 8 μgml^-1^. Besides, ophiobolin A lactone showed inhabitation effects against two pathogenic bacteria *Acinetobacter baumannii* and *E. faecalis,* with MIC values of 1 and 8 μgml^−1^, respectively^35^.

Generally, the crude extract activity against all microbes in nutrient agar was higher than their activity at the blood and Sabouraud dextrose agar, which could be to the complexity of the latter media resulting in the fungal interference chemical compounds and chemical composition of the media.

The cytotoxicity screening of fungal ethyl acetate extract improves approaches to ascertain the level of safety at which fungal extract could be applied. It was observed that *Chaetomium* sp. extract had activity against MDA-MB-231, HCT8, and KAIMRC1 cell lines with IC_50_ of 23.61 and 75.12 and 8.7 μgml^-1^, respectively. A similar observation trend was noted from *Chaetomium globosum* JN711454 crude ethyl acetate extract that exhibited cytotoxic effect against HepG-2, UACC62, MCF-7, and TK10 cells in 55, 43, 38, and 25% cytotoxicity, respectively^59^. Potential anticancer activity in a dose-dependent style was noted for *Chaetomium cupreum* extract against MCF-7 cell lines^60^. Chaetomugilins A, C, and F isolated from *Chaetomium globosum* originated from *Mugil cephalus* showed considerable inhibition against 39 human cancer cells^61^. Furthermore, a study by Li et al.^62^ approved high cytotoxicity against HepG2 human cell lines for azaphilone alkaloids and three other compounds isolated from *Chaetomium globosum* TY1, and cytotoxic effect ranged from 1.7 to 53.4 μM as IC_50_. Cytotoxicity of different compounds identified from *Chaetomium globosum* 7951 was evident against MCF-7, MDA-MB-231, HCT-8 cells growth as well as human lung cancer cell^63^. The extract from *Fusarium* sp. showed potential cytotoxic ability against all tested cell lines. In a similar observation, substantial cytotoxicity of ethyl acetate extract of *F. solani* isolated from *Datura metel* was noted against cervical cancer cells HeLa that induced cell apoptosis via mitochondrial pathway^64^. Majoumouo et al.^65^ investigated the apoptotic cytotoxicity for the extract of *Fusarium oxyporum* isolated from *Terminalia catappa* against human foreskin fibroblast and cervical cancer cells. The extract showed IC_50_ of 33.35 μgml^−1^ against cervical cancer cells; however, lower activity was noted against normal human foreskin fibroblast cells. This study approved a good cytotoxic potential of *Bipolaris* sp. against HCT116 and MDA-MB-231 cell lines. In accordance, the application of sulforhodamine B (SRB) assay for a cochlioquinone core compounds isolated from *Bipolaris sorokiniana* A606 showed powerful cytotoxicity against HepG-2, NCI-H460, SF-268 and MCF-7 tumor cell lines^66^. However, Tapfuma et al.^38^ approved no cytotoxic effect of *Bipolaris* sp. KTDS5 against UMG87 glioblastoma and A549 lung carcinoma cell lines contradicting results corresponding to tested cell variations. Radicinol and hamigerone compounds were identified from *Bipolaris papendorfii* and tested against cancer cell lines (IC_50_, 1.9 μM to 4.3 μM). Interestingly, the authors didn’t record toxicity for isolated compounds against the normal breast epithelial cell line suggesting their specificity in abnormal growth^67^. Ophiobolins and cochlioquinones were isolated from *Bipolaris oryzae*, the anhydrocochlioquinone A 95 activity against HeLa and KB cells^68^.

The biological activities of the crude fungal extracts could be mainly related to identified compounds that approved cytotoxic and antibacterial effects previously, although it can be isolated from different sources. Cochliodone A was recently isolated from *Chaetomium* sp. NA-S01-R1 cultured from deep sea showed cytotoxic effect against Hep G2 cell and antimicrobial ability against different *Vibrio sps.* and methicillin-resistant *S. aureus,* although weaker effect than the positive controls tested^63^. The study by Nakazawa et al.^69^ proposed the synthetic pathway of cochliodone A and chaetoglobin A where started by acetyl-CoA and involve CHGG_10027, further ammonification of cochliodone A might generate chaetoglobin A in a nonenzymatic reaction^70^. Furthermore, cytochalasins H and J and 18-metoxycytochalasin J were isolated from *Phomopsis* sp. from *Garcinia kola* and biologically tested. Activity against cervical cancer (LC_50_ = 3.66–35.69 μgml^-1^) and interestingly low toxicity was noted for normal cells, and high sensitivity was pointed out by some multi-drug-resistant microbes^71^. A halotolerant *Aspergillus* sp ethyl acetate extract provided Rosellichalasin, and Cytochalasin E compounds that showed potential cytotoxic effects against different cell lines such as RKO, BEL-7402, and A-549, providing IC_50_ ranged from 3.3–78 μM^72^.

On the other hand, four cytochalasins (2–5) and scoparasin C was identified from *Eutypella scoparia* PSU-H267 broth culture. Scoparasin C and cytochalasins 3 and 4 showed potential activity against Vero cell line^73^. Further cytochalasin alkaloids, Chaetoglobosins was previously isolated from *Chaetomium globosum*^74^. From the previous findings, it was found that a new class of dimeric product isolated from the fungus *Chaetomium globosum* cytochalasins with different oxidation outcomes in the macrocyclic portion. Compounds 3–6 contain a vinyl carbonate group of interest at C21 within the thirteen-membered macrocycle fused to an isoindolone bicyclic scaffold. Other large cytochalasin family members are less oxidized at the corresponding carbonate carbon than 1–4, including esters such as rosellichalasin and ketones as in cytochalasin G^75^. *Chaetomium elatum* ChE01 also presented chaetoglobosin V and six other chaetoglobosins (B-D) that had activity against cell lines of breast cancer and cholangiocarcinoma, providing IC_50_ ranged between 2.54–86.95 μM^76^. A*spergillus fumigatus* (AF3-093A) crude extract provided chaetoglobosin A and chaetoglobosin B.^77^. Such compounds showed antibacterial ability against *S. aureus*, and *Mycobacterium tuberculosis* H37Ra. Such fungal strain is rich in chemical compounds identified; however, it was not expected to have low antimicrobial ability which could be related to test conditions and strain tested. Furthermore, 25-hydroxyophiobolin I, 1β-hydroxy momilactone A as well as bipolatoxins A–F were recently isolated from *Bipolaris* species TJ403-B1. Sensitivity of *E. faecalis* was noted against bipolatoxin D with 8 μg/mL MIC^35^. Approved abilities of such biomolecules might conclude the different biological abilities of fungal extracts tested, although biomolecules from *Fusarium* were not fully identified, and furtherinvestigation is needed.

## Conclusions

This investigation was undertaken to develop fungal-based antimicrobial and cytotoxic agents. The fungal ethyl acetate crude extracts provided a significant antibacterial and dose-dependent style cytotoxic effect against some breast and colorectal cancer cell lines.

Promising antibacterial activity against all tested microbes was noted for *F. venenatum* extract when tested in nutrient agar. Furthermore, such fungal extract was also highly active against four cell lines where it is activity against HCT8 cell lines was almost similar to that of the positive control. On the other hand, the breast cancer cell line (MDA-MB-231) was sensitive to the three fungal extracts tested. The ability of the fungal extract to produce active ingredients against microbes and cancer cells was approved by the metabolic profiling of the fungal extracts. However, further studies are needed to isolate pure biomolecules from the different fungal extracts and test their antibacterial and cytotoxic potential and understand their action mode. Such an approach might result in the development of fungal-based agents for infection treatment.

## Supplementary Information

Supplementary Information 1.
