# Supplementary material for: Investigation of biological activity of soil fungal extracts and LC/MS-QTOF based metabolite profiling
Source: Sci Rep. 2021 Feb 26;11:4760. doi: 10.1038/s41598-021-83556-8 (PMC7910297; doi:10.1038/s41598-021-83556-8)
Supplement: Supplementary file 1 — Supplementary Information 1. [file 41598_2021_83556_MOESM1_ESM.docx]

**Investigation of Biological activity of soil fungal extracts and LC/MS-QTOF based metabolite profiling**

Afrah E. Mohammed^a*^, Hana Sonbol^a*^, Suaad Alwakeel^a*^, Modhi Alotaibi^a*^, Sohailah Alotaibi^a^, Nouf Alothman^a^, Rasha Saad Suliman^b,c^, Hanadi Talal Ahmedah^d,^ and Rizwan Ali^e^

| **1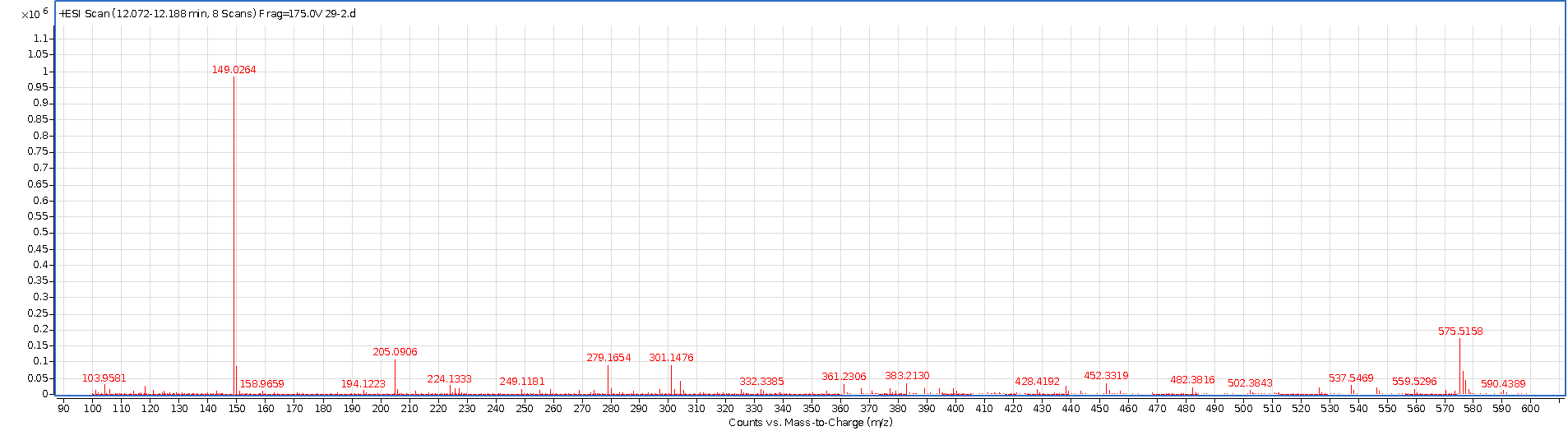** | **2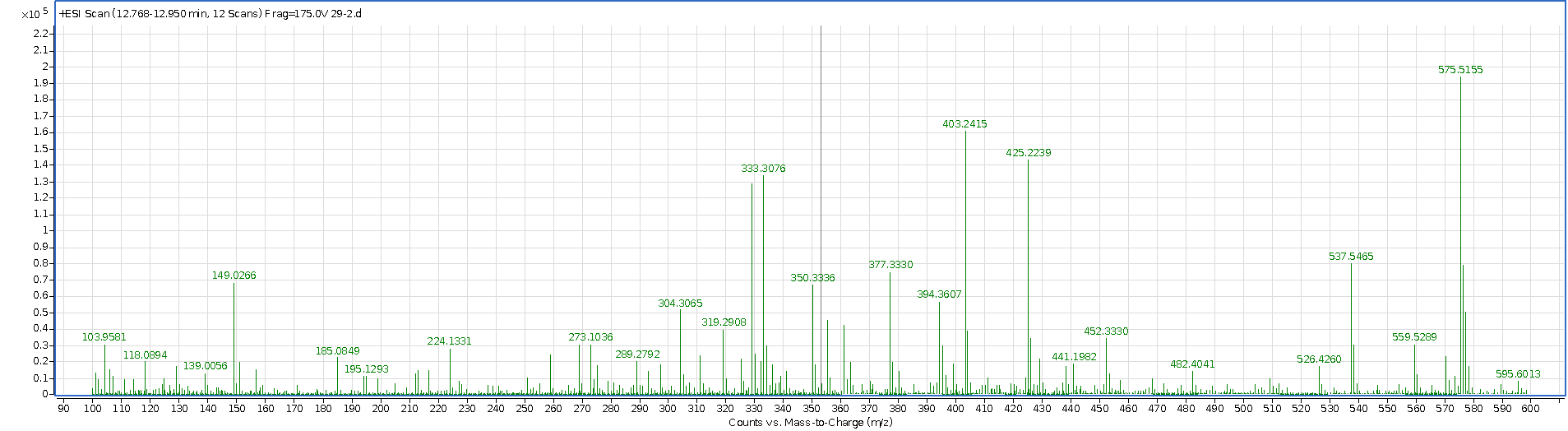** |
| --- | --- |
|  |  |
| **3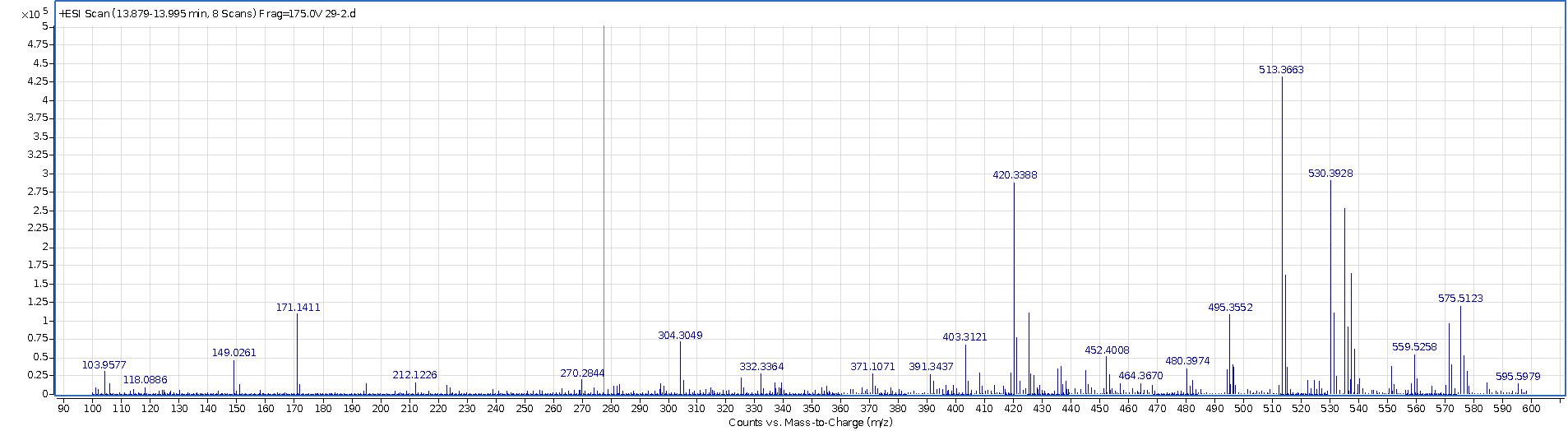** | **3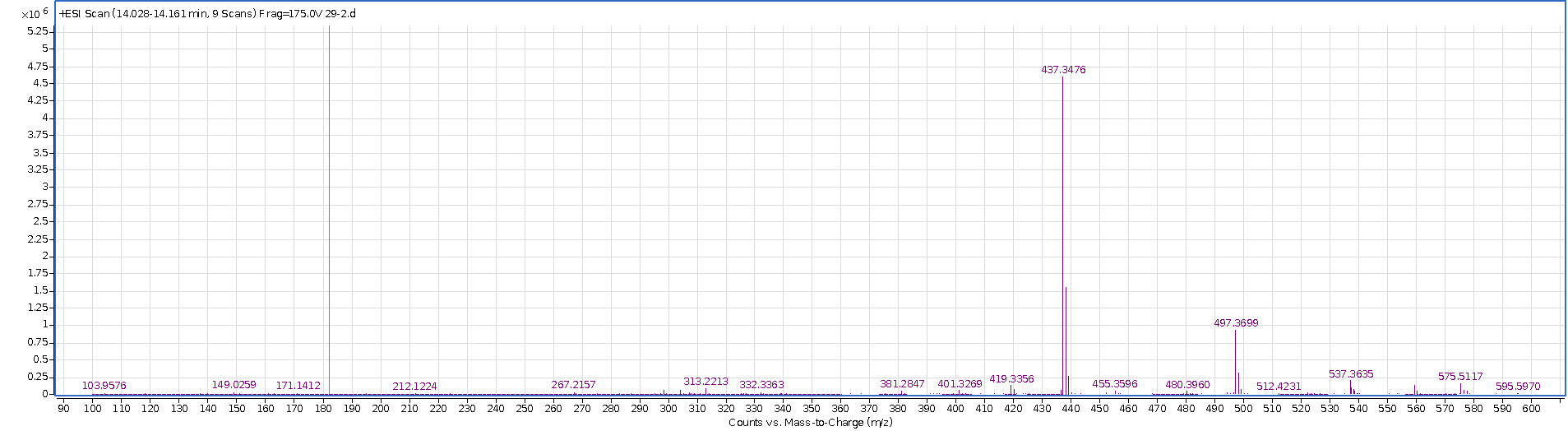** |
| **5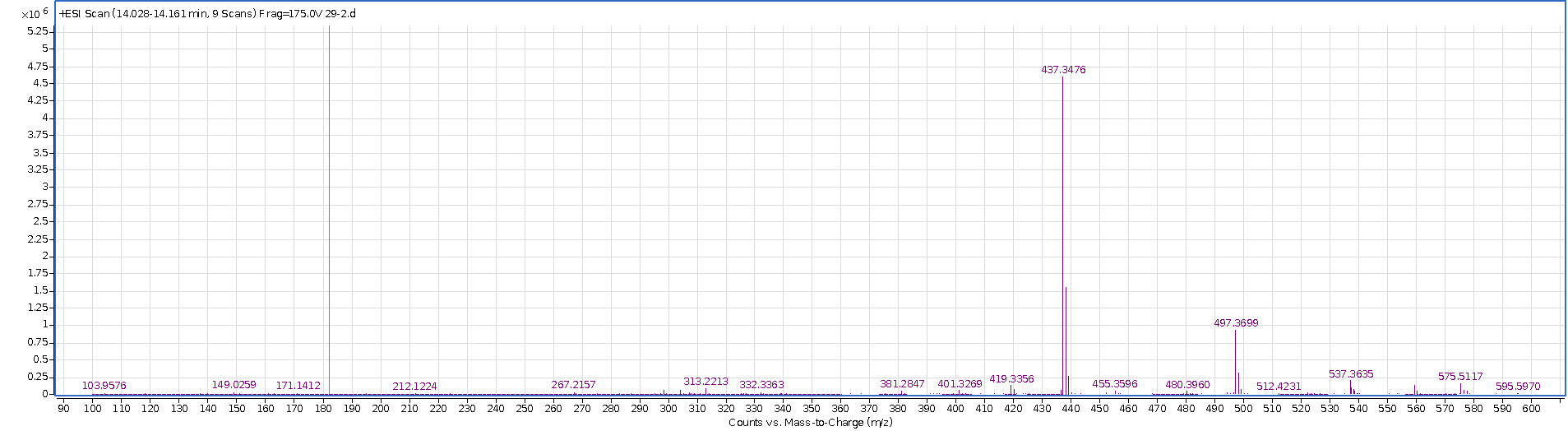** | **5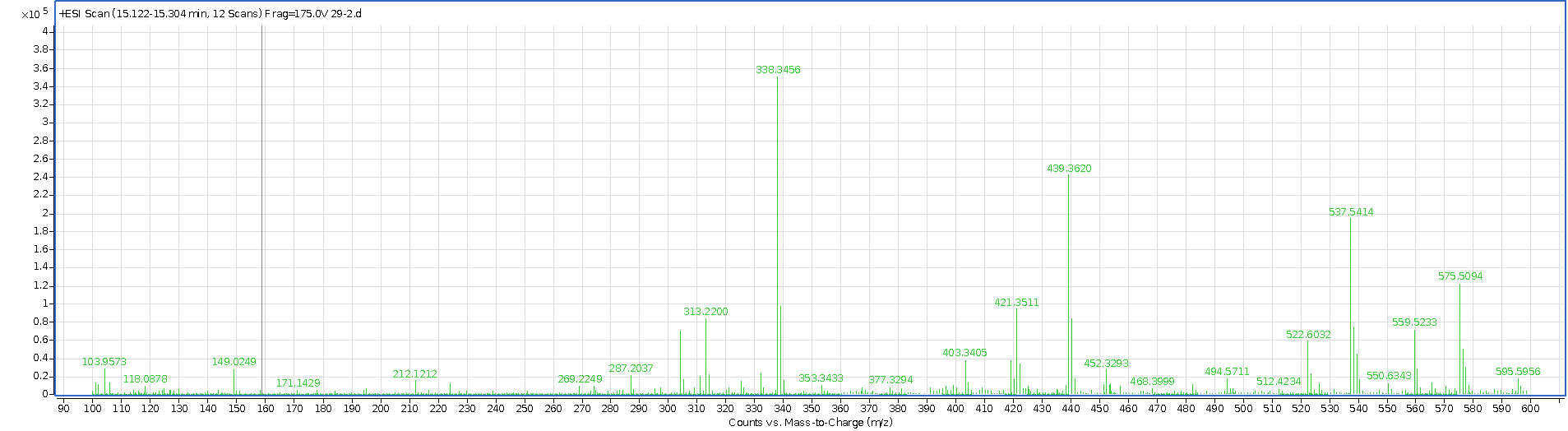** |
| Supplementary 1: Metabolomic profiling using LC–MS, chromatogram of ***F. venenatum*** extract presenting the molecular mass for different compounds | |
